# Advances in Platelet-Dysfunction Diagnostic Technologies

**DOI:** 10.3390/biom14060714

**Published:** 2024-06-17

**Authors:** Inkwon Yoon, Jong Hyeok Han, Hee-Jae Jeon

**Affiliations:** 1 Department of Smart Health Science and Technology, Kangwon National University, Chuncheon 24341, Republic of Korea; 2 Department of Mechanical and Biomedical Engineering, Kangwon National University, Chuncheon 24341, Republic of Korea

**Keywords:** platelet function test, platelet aggregation, hemostasis, clotting, point-of-care testing

## Abstract

The crucial role of platelets in hemostasis and their broad implications under various physiological conditions underscore the importance of accurate platelet-function testing. Platelets are key to clotting blood and healing wounds. Therefore, accurate diagnosis and management of platelet disorders are vital for patient care. This review outlines the significant advancements in platelet-function testing technologies, focusing on their working principles and the shift from traditional diagnostic methods to more innovative approaches. These improvements have deepened our understanding of platelet-related disorders and ushered in personalized treatment options. Despite challenges such as interpretation of complex data and the costs of new technologies, the potential for artificial-intelligence integration and the creation of wearable monitoring devices offers exciting future possibilities. This review underscores how these technological advances have enhanced the landscape of precision medicine and provided better diagnostic and treatment options for platelet-function disorders.

## 1. Introduction

Platelets play a vital role in managing bleeding and facilitating wound repair, acting as essential components in the regulation of blood clotting [1,2,3]. These tiny cell fragments circulate throughout the bloodstream and rapidly accumulate at injury sites to prevent hemorrhaging and promote healing [4,5]. Beyond their critical function in hemostasis, platelets contribute significantly to various other physiological processes, including inflammatory and immune responses, highlighting their comprehensive and indispensable roles in maintaining human health [5,6,7]. Reduced platelet function can result in a spectrum of disorders, ranging from mild to life-threatening ones. Such dysfunctions pose significant clinical challenges, particularly in the diagnosis and treatment of bleeding disorders. For instance, von Willebrand disease highlights the complex relationship between platelets and other clotting factors [8,9], while heparin-induced thrombocytopenia showcases the intricacies of platelet-related pathologies [10,11].

**Figure 1:**
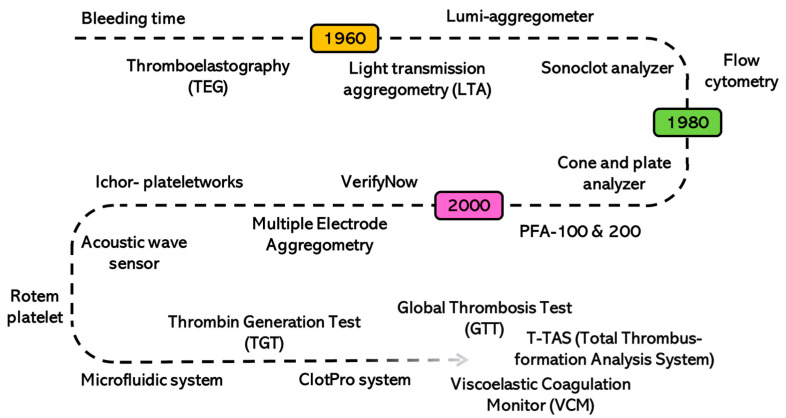
Timeline of developments in advanced platelet function test.

Advancements in platelet-function testing, as shown in Figure 1, have been transformative over the years, marking a significant shift from earlier qualitative evaluations to precise and measurable techniques [12,13,14]. Traditional methods, such as the bleeding time (BT) test, provide an initial look into the clotting efficiency, but lack the depth to fully capture the complexities of platelet behavior [15,16,17]. The development of platelet-aggregation tests, notably advanced by pioneers such as Dr. Gustav Born, has equipped the field with sophisticated tools to explore the detailed aspects of platelet functionality [3,18]. This paradigm shift has gained momentum with the integration of cutting-edge technologies into platelet diagnostics. Contemporary diagnostic tools, including flow cytometry and genomic sequencing, provide unparalleled insights into platelet function and the genetic factors linked to platelet disorders [19,20,21]. These advancements not only deepen our understanding of the underlying pathophysiology but also open avenues for targeted, personalized treatments [22]. The investigation of the evolution of platelet-function testing has revealed a vital intersection between technological progress and deepened knowledge in platelet science.

This review outlines the advancements in platelet-function testing, marking a significant evolution from traditional methods to cutting-edge technologies that are poised to revolutionize hematology. It emphasizes the crucial role of platelets in hemostasis and explores various diagnostic technologies, including microfluidic systems, digital microfluidics, and optical and acoustic wave sensors, for investigating platelet disorders and blood clotting. This article also discusses the impact of high-throughput sequencing (HTS) on enhancing clinical practices and the potential of these technologies to drive future innovations in the study and treatment of platelet dysfunction. In addition, we aimed to illustrate their impact on current clinical practice and their potential to foster innovations in hematology. This overview highlights advancements in our understanding of platelet disorders and personalized treatments. It also addresses the challenges of new technologies such as data interpretation and cost. Furthermore, we examine the potential of artificial intelligence and wearable devices in improving precision medicine and treatment options for platelet function disorders.

## 2. Current Platelet-Function Tests

### 2.1. Bleeding Time (BT) Test

Once the standard initial test for assessing primary hemostasis is completed, the BT test measures the duration of bleeding after a standardized skin incision [23]. Despite its historical significance, BT has largely been abandoned because of its poor sensitivity and specificity as well as significant operator-dependent variability [24]. The BT test is a clinical assessment used to evaluate primary hemostasis, specifically the time it takes for small blood vessels to stop bleeding after a controlled incision [25]. As listed in Table 1, there are several methods for performing a BT test, such as the Ivy method. This traditional method involves inflating a blood pressure cuff on the upper arm to a standardized pressure and making a small standardized incision on the ventral side of the forearm [26]. Blood is clotted at regular intervals, and the time from the incision to the cessation of bleeding is measured. Normal values for the Ivy method typically range from 2 to 7 min, and the test is terminated at 20 min if the bleeding continues [27]. Duke’s method is a less common method in which a small prick, usually on the earlobe or fingertip, is made using a lancet. The BT is recorded as the duration it takes for the bleeding to stop, with the usual time being approximately 2–5 min [28]. However, this method cannot be standardized and may result in the formation of a large hematoma at the puncture site.

### 2.2. Platelet Aggregation Test: Light Transmission Aggregometry (LTA)

**Figure 2:**
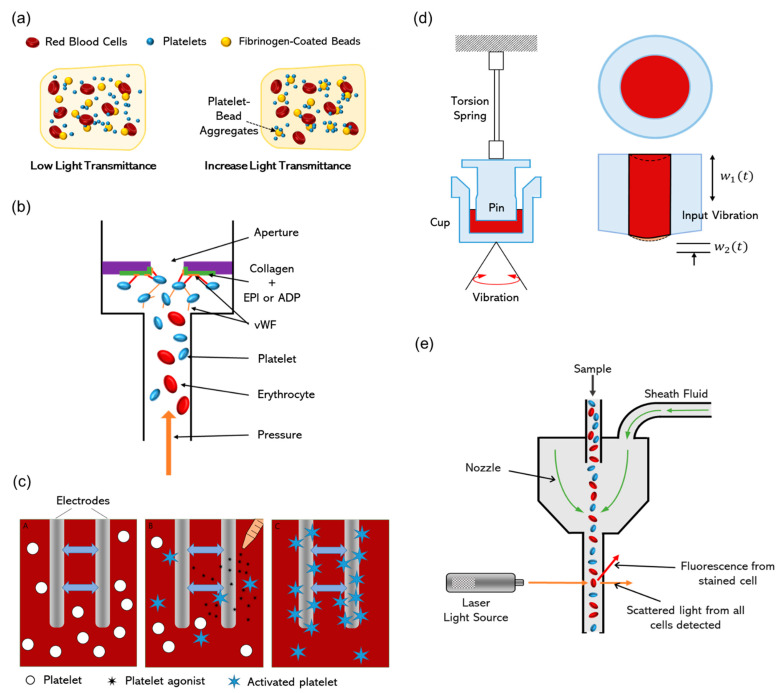
Comparative illustrations of platelet-function testing techniques. (**a**) VerifyNow test for measuring platelet reactivity; (**b**) platelet adhesion and aggregation with platelet function analysis (PFA) devices; (**c**) impedance aggregometry: measuring platelet aggregation through electrical resistance; (**d**) viscoelastic assessment of coagulation: TEG technologies; (**e**) platelet activation and population analysis via flow cytometry.

LTA is the gold standard for platelet-function testing and quantifies the aggregation capacity of platelets in response to various agonists [29]. The foundational principle of LTA relies on the optical changes that occur in platelet-rich plasma (PRP) as platelets clump together (Figure 2a). Initially, the turbidity of PRP is high because the suspended platelets scatter light. When an agonist, such as ADP, collagen, or epinephrine is added, platelets are activated and aggregate, leading to a reduction in turbidity [30]. As the aggregates become larger, they fall out of the suspension, clearing the plasma and increasing light transmittance. The change in light transmission is measured over time and has been determined to be directly proportional to the extent and rate of platelet aggregation [31]. This process is precisely monitored and recorded to yield a detailed trace that characterizes platelet function [32].

Recently, two significant advancements have been made in the LTA procedure. First, researchers have attempted to automate certain steps in the procedure [33]. This is exemplified using the Sysmex CS-2 × 00 series, which enables automated LTA without the need for highly experienced personnel [13,34,35]. This system automates steps such as the selection of agonists and their concentrations. Although it requires a smaller volume of platelet-rich plasma than traditional methods (140 µL vs. 200–500 µL), it incurs higher costs for reagents and consumables. However, it offers advantages in terms of labor and the ability to generate concentration–response curves more efficiently [35]. The second development is the potential for these automated assays to be conducted in non-specialist centers, with the results being forwarded to tertiary centers for expert analysis. This could help standardize the assay and improve reproducibility across different laboratories worldwide, despite the continued need for expert interpretation of the results [36].

The third significant development was the introduction of high-throughput 96- to 384-well plate assays, which offer a more comprehensive analysis of platelet function in less time and with less plasma [37]. These plate-based assays can run multiple tests simultaneously, facilitating the creation of detailed concentration–response curves for various agonists. However, their complexity may challenge their interpretation [38]. Currently, these assays are mainly confined to research settings. While they provide a faster, more efficient way to screen for platelet-function disorders, they are not intended to replace traditional LTA. Nevertheless, they could serve as a preliminary screening tool outside specialized centers. In 2021, Kim et al. [39] developed a centrifugal microfluidic device designed to enhance the efficiency and accuracy of platelet-function testing. This device operates on the principle of centrifugal force, where blood samples are driven through microfluidic channels to specific testing chambers by the force generated from rotational movement. As the device spins, different reagents that trigger platelet activation are introduced at calibrated intervals, allowing for the simultaneous assessment of multiple aspects of platelet function under dynamic flow conditions. This method not only reduces the sample volume to less than 1 mL but also speeds up the testing process and reduces potential errors associated with manual sample handling.

In addition to the innovative approaches discussed, the widely used VerifyNow system is an essential point-of-care tool in platelet-function diagnostics [40,41]. It measures platelet-induced aggregation in response to specific agonists impregnated on fibrinogen-coated test cartridge beads, effectively mimicking in vivo platelet–fibrinogen interactions (Figure 2a) [42]. The VerifyNow system is particularly valuable in managing patients undergoing anti-platelet therapy, offering distinct assays for those receiving aspirin, clopidogrel, or other P2Y12 inhibitors. By providing rapid, quantitative measurements of platelet reactivity, the device helps clinicians make data-driven decisions about adjusting medication dosages or switching therapies to optimize patient outcomes [43].

The system quantifies platelet function through measuring changes in light transmittance as platelets aggregate in the sample. Its use of whole blood eliminates the need for sample preparation, while its standardized, cartridge-based design ensures reproducible testing across various clinical settings, from cardiology units to outpatient clinics [44]. The VerifyNow system’s design accurately reflects in vivo hemostatic processes, offering insights into platelet–fibrinogen dynamics that closely mirror physiological events. This, combined with its rapid turnaround time and high reproducibility, makes VerifyNow an indispensable tool for managing anti-platelet therapy, particularly in urgent clinical situations.

### 2.3. Platelet Adhesion in Platelet-Function Analyzers

In assessing platelet function within environments that closely mirror physiological conditions, both the platelet function analyzer (PFA) series and the cone and platelet analyzer (CPA) provide unique and complementary perspectives [45]. PFA devices, including the PFA-100 and PFA-200, were designed to mimic platelet adhesion and aggregation in response to vascular injury [46]. This emulation is achieved by drawing blood through cartridges fitted with membranes coated with collagen and other agonists, simulating the high shear stress typical of arterial blood flow and exposure of the subendothelial matrix following vessel wall damage [47]. The key metric provided by these devices—the closure time—is the interval required for platelets to form a plug and occlude the membrane aperture, offering quantifiable insight into platelet adhesion, activation, and aggregation under physiologically relevant shear conditions (Figure 2b) [48].

By contrast, the CPA (Cone and Platelet Analyzer) adopts a distinct approach through placing a small volume of anticoagulated blood under uniform shear stress induced by a rotating cone over a plate [44]. This process is achieved without the introduction of biomimetic surfaces, focusing instead on the natural ability of platelets to adhere and form aggregates [45]. The extent of these aggregates or the surface area they cover serves as a reliable indicator of platelet functionality under shear conditions, mimicking those found in smaller arterioles and capillaries. These technologies do not analyze traditional clot formation but rather assess the critical aspects of platelet dynamics such as adhesion, activation, and aggregation, under conditions that simulate the physiological flow of blood. Their combined application is crucial, not only for assessing bleeding disorders and the efficacy of anti-platelet treatments, but also for enhancing the diagnostic evaluation of platelet functions rather than general clot formation processes. Thus, they significantly advance our understanding of platelet function in both clinical and research settings [46].

### 2.4. Impedance Aggregometry

Impedance aggregometry is a fundamental technique for assessing platelet function that harnesses the principles of electrical conductance to provide critical insights into platelet aggregation capabilities [49]. At the heart of this methodology is the impedance analyzer, a device that quantifies the electrical resistance (or impedance) across electrodes submerged in either platelet-rich plasma (PRP) or whole blood [50]. The introduction of specific agonists initiates platelet activation, leading to the formation of aggregates that impede electrical current and elevate impedance (Figure 2c). This impedance shift is directly proportional to the platelet aggregation level, offering a quantifiable reflection of platelet functionality [51]. A significant advantage of impedance aggregometry is its compatibility with whole blood assays, which better replicate the physiological environment of platelets and ensure a more accurate depiction of their function in vivo. Additionally, the simplicity of this technique and minimal equipment requirements make it ideal for point-of-care settings, facilitating rapid and effective clinical assessment [52]. The standard procedure encompasses sample preparation, analyzer setup, agonist introduction for platelet activation, continuous impedance monitoring, and the subsequent analysis and interpretation of these measurements to evaluate platelet aggregation [53].

To enhance this methodological framework, a multiplate analyzer introduces advanced capabilities to the domain of impedance aggregometry [54]. This sophisticated instrument automates the measurement of impedance changes due to platelet aggregation, enabling detailed analysis of platelet behavior in response to a variety of agonists. Its capacity for multiple simultaneous tests allows the extensive assessment of platelet reactivity, enriching the diagnostic process with comprehensive data [55,56]. The key features of the multiplate analyzer, such as multichannel testing, automated data analysis, compatibility with both PRP and whole blood, and user-friendly interface, underscore its value in clinical diagnostics. These attributes streamline the diagnostic process, ensure the physiological relevance of the tests, and facilitate rapid decision-making in clinical settings. Particularly beneficial in point-of-care applications, the multiplate analyzer is an essential tool for the management of clotting disorders, monitoring of anti-platelet therapy, and evaluation of bleeding risks, thereby amplifying the clinical utility of impedance aggregometry owing to its advanced technology and operational ease [57,58].

### 2.5. Viscoelastic Properties in Clot Assessment

Thromboelastography (TEG) and rotational thromboelastometry (ROTEM) are innovative viscoelastic testing technologies that have significantly advanced the diagnosis and management of platelet dysfunction through providing a detailed and comprehensive overview of the clotting cascade [59]. These methods assess the viscoelastic properties of blood and provide real-time insights into the kinetics of clot development, stabilization, and dissolution, which are crucial for understanding platelet function. The principle underlying both TEG and ROTEM involves the placement of a whole blood sample in a cup, which is then subjected to rotational or oscillatory movements (Figure 2d). This motion facilitates physical changes associated with blood clotting, which are subsequently translated into a graphical trace [60]. This trace delineates various critical parameters of clot formation, such as the time to clot initiation (reaction time), kinetics of clot formation, clot strength, and fibrinolysis. The clot strength, reflected by the amplitude of the trace, is strongly correlated to the functionality and number of platelets, illustrating their pivotal roles in interacting with fibrinogen and other clotting factors to fortify the clot [61]. Furthermore, TEG’s Platelet Mapping™ assay can measure the reduction in platelet function due to anti-platelet therapies like aspirin and clopidogrel, providing valuable insights for tailoring treatment to individual patient needs. Thus, TEG and ROTEM not only illuminate the qualitative functionality of platelets within the clot-formation process, but also provide a holistic view of hemostasis, encompassing fibrin formation, platelet contribution, and the breakdown of clots through fibrinolysis [62].

Viscoelastic tests have become indispensable tools in the management of platelet dysfunction, particularly in clinical scenarios that require an intricate understanding of a patient’s coagulation profile, such as during significant surgical interventions or the treatment of trauma-induced bleeding [63]. By providing approaches to evaluate the entire clotting process, TEG and ROTEM enable clinicians to tailor therapeutic strategies more effectively and ensure optimal patient care. However, despite their significant impact on clinical diagnostics and patient management, the widespread adoption of TEG and ROTEM has been limited by several factors [64]. The high cost of equipment and the need for specialized training to accurately interpret the complex data they generate limit their application to well-equipped hospital settings and specialized care centers. These limitations highlight the balance between the advanced diagnostic capabilities offered by these technologies and the practical considerations for their implementation in diverse clinical environments [65].

### 2.6. Other Techniques

Beyond these techniques, a suite of advanced techniques—plateletworks, the global thrombosis test (GTT), and SonoClot Analyzer—broadens the scope of platelet function and coagulation analysis. Each of these methodologies offer a unique lens for viewing the complex dynamics of hemostasis and provide invaluable insights for both the research and clinical management of coagulopathies [66,67,68].

Plateletworks employs the straightforward principle of directly comparing platelet counts before and after agonist-induced aggregation from whole blood samples [69]. Its rapid turnaround time and ease of use at the point-of-care make it especially useful in acute settings, in which the immediate assessment of platelet function is critical. However, its reliance on specific agonists limits the assessment of aggregation pathways triggered by these agonists.

The GTT evaluates the kinetics of thrombus formation and fibrinolysis under physiological flow conditions and provides a comprehensive overview of the clotting and clot-dissolution processes [70]. The ability of this test to reflect the integrated function of platelets, coagulation factors, and fibrinolytic activity in whole blood is a significant advantage. Specifically, it measures shear-dependent clot formation in non-anticoagulated blood, which allows for a more accurate representation of in vivo hemostasis. Nevertheless, the need for the precise control of flow conditions can pose practical challenges in routine clinical practice [71].

Meanwhile, the Sonoclot Analyzer measures the viscoelastic properties of blood during clot formation and dissolution, offering real-time insights into clot kinetics. This device is particularly adept at detecting changes in the blood’s physical properties as it clots, thereby providing critical information about platelet function and fibrinolysis [72]. Unlike TEG, which primarily assesses clot initiation and strength, Sonoclot emphasizes the interactions between platelets and fibrin as well as the process of clot retraction. This focus makes it particularly valuable for evaluating platelet functionality within the clotting process. To further enhance its utility in platelet studies, specialized protocols can be implemented with the Sonoclot Analyzer. For instance, the use of platelet-specific reagents, such as ADP or collagen, can be introduced during the test to trigger and measure platelet activation and aggregation directly. Additionally, modifications to the standard test protocol can include varying the concentration of these agonists to assess the responsiveness of platelets under different conditions, providing a more nuanced understanding of platelet behavior in various pathological states. Such protocols are instrumental in identifying subtle dysfunctions in platelet activity that may not be evident under normal testing conditions [73,74].

Collectively, these techniques expand the diagnostic toolkit available for evaluating platelet function and the clotting process. Each offers distinct advantages, which include the rapid point-of-care assessment capabilities of Plateletworks, the comprehensive hemostatic evaluation using GTT, and the dynamic clot assessment offered with the Sonoclot Analyzer. Despite their varied applications and operational considerations, these technologies collectively enhance a clinic’s ability to diagnose, monitor, and manage a wide range of coagulopathies and platelet-function disorders, contributing significantly to the advancement of hemostasis research and clinical care [73,74].

**Table 1:** Comparison of platelet-function testing methods: advantages, disadvantages, and testing principles.

Categorize	Monitoring Types	Advantage	Disadvantage	Principle of Test	Ref.
Bleeding time test	Ivy	Physiological test, fast monitoring	High risk of scarring, risk of infection and bleeding, invasive poorly standardized, dependent on many variables	Creates a wound to assess platelet function through measuring the time taken for the bleeding to stop	[12,75,76]
	Duke				
	Template				
Platelet Aggregation Test	Light transmission aggregation (LTA)	High flexibility, high sensitivity to anti-platelet therapy, analysis of various platelet function disorder, investigation of various platelet pathways	Time-consuming, difficult to sample preparation, required skilled laboratory personnel, pre-analytical and procedural variability	Measures platelet aggregation in blood through tracking changes in light transmission as platelets clump together. (LTA uses PRP, VerifyNow uses whole blood).	[41,75,77,78]
	VerifyNow	User-friendly, simple and rapid result generation	Inflexible, high cost, monitoring anti-platelet therapy, limited hematocrit (HCT) and platelet count		
	Lumi-aggregometry	Dual measurement, enhanced sensitivity, better diagnosis, therapy monitoring	Expensive, complex setup, time-consuming, skilled personnel	Measures both platelet aggregation and the release of adenosine triphosphate (ATP) form platelet dense granules	
Platelet Adhesion in Platelet-function Analyzers	Platelet function analyzer (PFA-100/200)	Whole blood test, required small blood volumes, simple and rapid, investigating severe platelet defects	Inflexible, HCT-dependent not sensitive to platelet secretion defects	Mimics platelet adhesion and aggregation under high shear stress in a membrane-coated system. Measures closure time	[78,79,80]
	Cone and platelet analyzer	Small blood volume, simple and rapid	Expensive, lack of clinical studies, required skilled laboratory personnel	Measures blood coagulation and platelet aggregation through applying shear stress through a rotating cone	
Electrical conductance	Impedance platelet aggregation	Flexible, no sample processing simple diagnostic method, sensitive to anti-platelet therapy	Insensitive, limited HCT and platelet count range	Uses impaired electrical conductance to assess platelet aggregation in whole blood	[55,56]
	Multiplate	Whole blood samples, simple and rapid, standardized activators, bedside monitoring	Susceptibility to interference from certain medications and substances		
Viscoelastic Properties in Clot Assessment	Rotational thromboelastometry (ROTEM)	Real-time monitoring	Measures clot properties only, lack of clinical studies, limited HCT and platelet count range	Evaluates the viscoelastic properties of blood during clotting to assess overall hemostasis, including platelet function. Similar to ROTEM, measures the viscoelastic properties of clot formation for a comprehensive assessment of coagulation and platelet function	[60,81,82]
	Thromboelastography (TEG)		Measures clot properties only, lack of clinical trial, low shear-induced		
Other techniques	Platetletworks	Low cost, small blood volume required, simple and rapid, platelet count measurement	Indirect test, scarce data	All methods analyze platelet function and blood coagulation processes to provide insights into thrombosis, coagulation, platelet activation, and platelet–blood interactions	[83,84]
	Global thrombosis test (GTT)	Whole blood samples, small blood volume required	Lack of clinical studies		[85,86]
	Sonoclot	Effective clinical hemostasis management comprehensive clot assessment, rapid output, POC convenience, versatility in sample types	Measures clot properties only		[87,88]

## 3. Advanced Technologies for Platelet-Function Testing

Platelet-function testing has undergone a significant transformation in recent years to counter the limitations of earlier methodologies [89,90]. Traditional platelet function tests, such as BT and LTA, face challenges in terms of reproducibility, sensitivity to platelet-function disorders, and the ability to replicate the physiological conditions of blood flow and shear stress [91,92]. These earlier tests frequently required larger blood volumes and time and did not provide real-time monitoring, limiting their utility in acute clinical settings [93,94]. Furthermore, genetic contributors to platelet disorders are largely underexplored due to the limitations of older genomic techniques. In response to these limitations, advanced technologies have been developed to address the need for precise, dynamic, and comprehensive platelet function-assessment tools [39,95,96].

### 3.1. Microfluidics and Lab on a Chip

Recent advancements in microfluidic platforms have underscored the capability of this technology to emulate the vascular environment, providing a dynamic platform in which blood can flow through microscale channels. Such a setup subjects platelets to shear stresses akin to those in human vessels, enabling researchers to observe platelet activation, adhesion, and aggregation in conditions that closely resemble those within the human body. Such systems, while offering a high-fidelity representation of in vivo conditions with the benefit of requiring only minimal blood volumes, face the challenges of design complexity and the need for specialized operational equipment, which limit their application in broader clinical settings [97,98].

**Figure 3:**
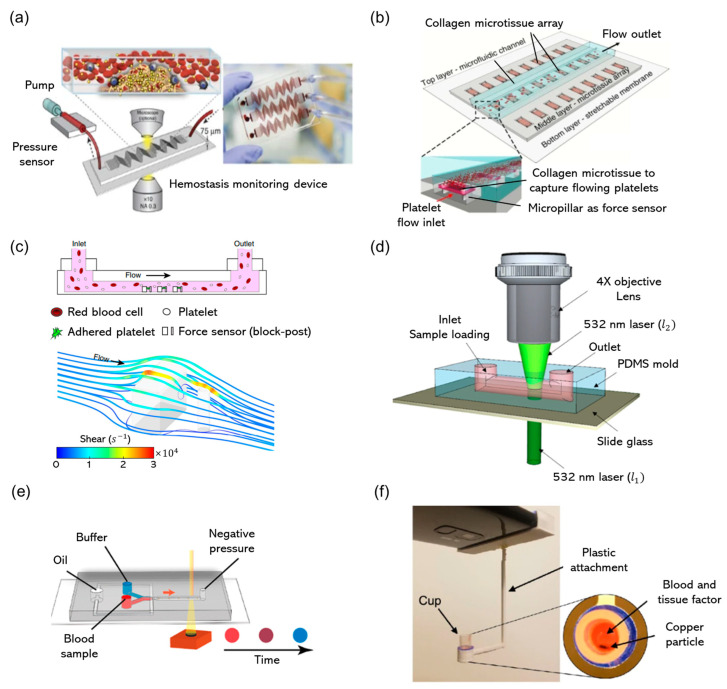
Innovative methods for assessing hemostasis and platelet function test. (**a**) Biomimetic microfluidic device for monitoring functionality of mimicking various vascular shear rate. Adapted with permission [94], copyright 2016, Springer Nature. (**b**) A layered microfluidic system using collagen to capture microclot. Adapted with permission [99], copyright 2019, Springer Nature. (**c**) Whole blood over microscale blocks and posts with computational simulations of shear rate. Adapted with permission [100], copyright 2019, Springer Nature. (**d**) Platelet-function test using speckle pattern monitoring system. Adapted with permission [97], copyright 2019, Springer Nature. (**e**) Viscosity-based coagulation test using droplet microfluidics and smartphone technology. Adapted with permission [101], copyright 2022, American Chemical Society. (**f**) Coagulation testing using smartphone vibration to agitate a copper particle in the blood sample. Adapted with permission [102], copyright 2022, Springer Nature.

Jain introduced a microfluidic device that simulates stenosed arteriolar vessels to assess blood clotting under specific flow conditions with small sample volumes (Figure 3a). This device, which integrated a mathematical model for clotting analysis, enabled accurate in vitro coagulation and platelet-function measurements and offered real-time coagulation monitoring in pig models for endotoxemia and heparin therapy [94]. While promising for personalized diagnostics and antithrombotic therapy monitoring, its complexity, need for specialized equipment, and validation for human clinical use, along with concerns regarding cost and environmental impact, posed challenges for its widespread adoption. Chen et al. [99] introduced the clotMAT system to measure clot formation under physiological conditions, integrating microfluidics with microclot array elastometry (Figure 3b). The clotMAT is composed of three functional layers: the top layer is made of PDMS with multiple microfluidic channels for platelet flow; the middle layer consists of PDMS embedded with arrays of collagen microtissues that capture flowing platelets and report clot contractile forces; and the bottom layer is a stretchable silicone membrane that measures clot stiffness. This technology marked a significant advancement in the study of hemodynamics and clot mechanics, particularly in understanding the effects of procoagulants, platelet antagonists, and plasma on clot stiffening in patients with bleeding disorders. Because it provides insights into the individual and combined effects of biochemical treatments and shear flow on clot formation, clotMAT can significantly impact research and diagnosis of bleeding disorders. However, a potential limitation or challenge of this technology is its complexity and the need for specialized equipment, which might limit accessibility for some laboratories. Additionally, the adaptation of the system to a wide range of clinical conditions and translation of its findings to direct clinical applications may require further validation and studies.

Kaikita et al. [103] introduced the Total Thrombus Formation Assay System (T-TAS), a microchip-based flow chamber system developed to assess thrombus formation in whole blood. T-TAS utilizes two primary types of microchips to analyze thrombus formation: the platelet (PL) chip and the atheroma (AR) chip. The PL chip is coated with type I collagen and primarily measures platelet aggregation to assess primary hemostatic ability. In contrast, the AR chip is coated with both type I collagen and tissue thromboplastin, measuring fibrin-rich platelet thrombus formation by activating the coagulation system alongside platelet aggregation. This system quantifies thrombus formation by calculating the area under the flow pressure curve (AUC). T-TAS is particularly useful for evaluating the therapeutic effects of various antithrombotic agents and is employed to assess the efficacy of anti-platelet therapy in patients with coronary artery disease and cerebrovascular disease. Additionally, compared to traditional methods of assessing platelet function, T-TAS provides a more comprehensive evaluation of primary hemostasis. By integrating the assessment of platelet aggregation and coagulation activation, T-TAS offers a holistic evaluation of the efficacy of antithrombotic therapies and serves as a critical tool for predicting bleeding and thrombotic risks in patients with various cardiovascular diseases [104].

Ting et al. [100] introduced a microfluidic method to rapidly measure the contractile force of platelets, which is important for assessing clot strength and detecting platelet dysfunction (Figure 3c). This technology proved effective in identifying the impact of anti-platelet therapy and predicting risk of hemorrhage in trauma patients. However, its reliance on specialized equipment and need for extensive validation are potential drawbacks that limit its immediate adoption in clinical practice. Jeon et al. [97] developed a novel platelet function-testing method using laser speckle contrast imaging in a microfluidic system. This approach quantified platelet aggregation through measuring the speckle decorrelation time (SDT) of blood flow. The results revealed clear differences in the SDT between whole blood and platelet-poor blood, with specific platelet agonists such as ADP, epinephrine, and arachidonic acid further influencing it. This suggests the utility of the method for instantly identifying the risk of bleeding and evaluating anti-platelet treatments using only a small blood sample (Figure 3d) [101]. Luna et al. [105] introduced a biomimetic microfluidic device designed to mimic stenosed and tortuous arteriolar vessels, enabling the analysis of blood clotting under flow conditions using only a small volume of blood. This device, when connected to a pressure sensor, can accurately measure coagulation, platelet count, and fibrin content in real time. Its potential in a clinical setting was demonstrated by using it to detect prolonged clotting times in blood samples from pediatric patients undergoing extracorporeal membrane oxygenation and receiving anticoagulants. Despite these advancements, the potential disadvantages may include the complexity of device operation, the potential for variability in clotting-time measurements across different patient conditions, and the need for integration into current clinical workflows. Chen et al. [101] developed a simplified microfluidic device for coagulation testing that used color changes in blood droplets to assess viscosity and clotting. Although this promised faster and less resource-intensive point-of-care testing, its challenges may include ensuring accuracy across varying clinical conditions and standardizing the device for widespread clinical use (Figure 3e,f). Chan developed a new smartphone-based method for testing prothrombin time (PT) and international normalized ratio (INR). Using the phone’s vibration motor and camera, the movement of copper particles was tracked to measure the PT/INR in blood samples. When tested against 140 plasma and 80 whole blood samples, the system exhibited high accuracy comparable to that of clinical analyzers. This affordable and accessible technology could revolutionize PT/INR testing, especially in low-resource settings (Figure 3f) [102].

Microfluidic technologies in hemostasis research have also advanced significantly, focusing on modeling complex vascular networks and blood-flow parameters to understand the pathophysiology of blood disorders. Various lab-on-a-chip devices for hemostasis-related disorders and antithrombotic therapies were developed using advanced design specifications, fabrication techniques, and diverse materials such as polydimethylsiloxane and thermoplastics. Despite these advances, microfluidics has several drawbacks, including the complexity and need for specialized equipment for operation and data interpretation, potentially restricting its use to specialized centers. There is also a challenge in translating the findings from these devices to clinical applications, which requires further validation. In addition, concerns have been raised regarding the cost and environmental impact of the disposability of some of these devices. Despite their high throughput and reduced sample-size benefits, these technologies still face limitations in terms of compatibility with all organic solvents and challenges such as microchannel deformation under high-pressure conditions, which can be problematic in certain applications.

### 3.2. Digital Microfluidics for Platelet Research

Digital microfluidics is a technique that manipulates small droplets of liquid at the microscale using electrical signals, which is ideal for precise, low-volume testing. In this technology, an electrical voltage is applied to an array of electrodes to mobilize, mix, split, or merge droplets through changes in the surface-wetting properties, a process known as electrowetting. In platelet research, digital microfluidics enable highly controlled experiments with minimal blood sample volumes, support high-throughput automated testing, and can be easily integrated with sensors for real-time data analysis. These attributes make it particularly useful for studying platelet–drug functions and drug interactions efficiently and accurately.

Sista et al. [106] developed a low-cost, electrowetting-based digital microfluidic platform for rapid and efficient point-of-care testing. It could perform a diverse set of assays, including immunoassays and PCR, in minutes, with only a small volume of blood sample. Despite its portability and cost-effectiveness, challenges included implementation complexity, reliability across various tests, and the need for specialized training. Cakmak et al. [107] developed a microfluidic coagulation testing cartridge using microelectromechanical systems (MEMS) technology for rapid and accurate aPTT and PT tests using minimal plasma. The system’s innovative nonelectrical connection reduces reliability issues. Nevertheless, it still faces challenges such as operational complexity, manufacturing consistency, and integration into clinical workflows. Simplification and cost reduction are key areas for future enhancements to promote wider clinical adoption.

Li et al. [108] developed a novel point-of-care blood coagulation assay using printed circuit board (PCB)-based digital microfluidic devices. This assay measured the clotting tendency and stiffness by tracking the movement and deformation of a blood drop across a PCB electrode array, with results validated against standard coagulation tests. While offering faster and less sample-consuming tests with high clinical relevance, the potential drawbacks include the need for the precise calibration of hardware and software for accurate image analysis and velocity tracing, the complexity of integrating PCB technology into clinical settings, and the need to ensure the reliability of the device across a wide range of blood conditions. Innovations in digital microfluidics have opened new avenues for platelet research, with He et al. [109] utilizing this technology to explore the effects of various anti-platelet agents at a previously unattainable precision level. By manipulating tiny droplets on a hydrophobic surface using electrokinetic forces, researchers can conduct high-throughput screening and detailed studies of platelet behavior, ushering in personalized medicine. However, the advanced nature of this technology, which requires a sophisticated setup and specialized knowledge, may restrict its application to specialized research settings, rather than routine clinical use.

Qin et al. [110] developed a method using digital microfluidics to assess the platelet aggregation rate, offering precise control over small liquid volumes and thus improving on traditional techniques that require larger samples and struggle with low aggregation rates or chyle blood. While promising for the enhanced diagnosis and treatment of platelet dysfunction, the challenges of this method include setup costs, training requirements, and ensuring method reliability and accuracy in the clinical environment. Despite this innovation, digital microfluidics faces challenges such as integration complexity, potential cross-contamination, and scalability issues. Therefore, further advancements could make it a key piece of technology in diagnostics and biological research.

### 3.3. Flow Cytometry

Flow cytometry is a core technique for platelet phenotyping that offers insights into the platelet activation status through analyzing activation marker expression, unlike aggregometry methods that focus on platelet aggregation dynamics (Figure 2e). Studies comparing LTA with flow cytometry for the detection of inherited platelet disorders have demonstrated the former’s advantages, such as lower blood volume requirements and no need for platelet-rich plasma preparation. With a negative predictive value of 87%, flow cytometry can serve as an initial screening test before employing LTA to provide complementary information on platelet-function defects. However, its application in diagnostics requires further validation and standardization, particularly concerning platelet activation thresholds, fluorescence intensity levels, agonist types and concentrations, and the use of fixatives [111,112,113].

Additionally, the mepacrine assay in flow cytometry, which assesses platelet δ-granule incorporation and secretion, can help exclude platelet dense granule deficiency. However, similar to other assays, it faces standardization challenges, including variations in mepacrine concentration and incubation conditions. The measurement of CD63 expression post-activation offers another method to evaluate δ-granules secretion, although it does not distinguish between storage pool deficiency and primary secretion defects. Combining this method with a mepacrine assay may improve the characterization of dense granule disorders. Flow cytometry has also been employed to detect phosphatidylserine expression in activated platelets to diagnose conditions such as Scott syndrome [114,115,116,117].

**Figure 4:**
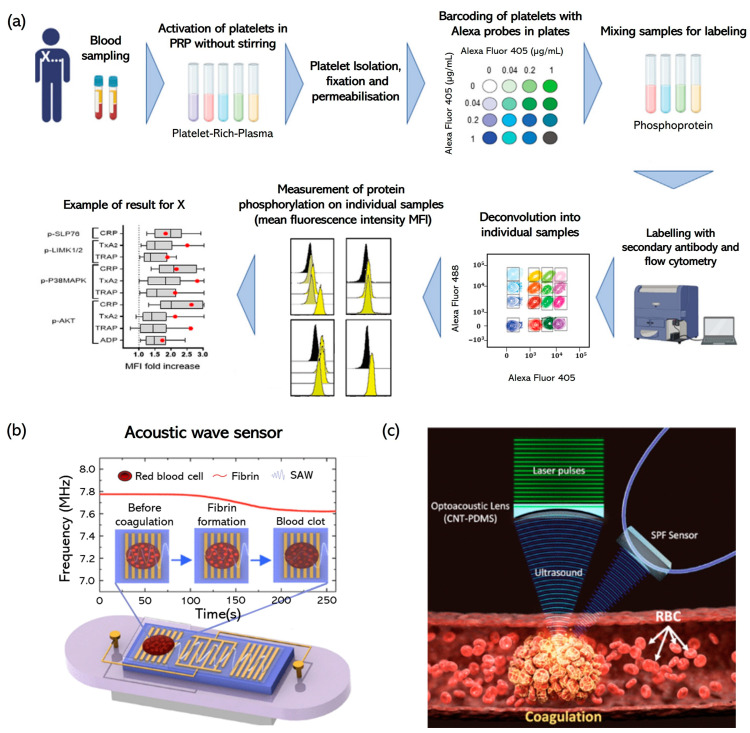
Advanced diagnostics in platelet function and coagulation disorders. (**a**) Multicolor flow cytometry using clinical samples. Adapted with permission [118], copyright 2023, Elsevier. (**b**) Novel Love-mode surface acoustic wave (SLSAW) sensor. Adapted with permission [119], copyright 2020, American Chemical Society. (**c**) Assessment of whole blood coagulation using the micro-ultrasonic and adaptive signal-processing algorithm. Adapted with permission [120], copyright 2022, American Chemical Society.

Garcia et al. [118] introduced a method that used multicolor flow cytometry with fluorescent barcoding to efficiently assess platelet signaling pathways (Figure 4a). Benefiting from advancements in cytometers and commercial antibodies, this technique enables the simultaneous examination of pathways activated by major platelet agonists in small blood volumes. Initial comparisons with immunoblotting helped select specific phosphoproteins and conditions for the study, which involved patients with unexplained minor bleeding, type-1 von Willebrand disease, and confirmed platelet disorders. The study discovered distinct patterns of phosphoprotein responses to various agonists, with clustering algorithms highlighting the unique responses of individuals, especially noting lower responsiveness in patients with platelet storage pool deficiencies. This approach is a fast and effective way to profile platelet signaling for clinical purposes, offering a significant supplement to functional tests.

## 4. Other Special Techniques for Platelet Analysis

The emergence of optical and acoustic wave sensors has introduced a novel approach for the noninvasive and real-time monitoring of platelet function [121,122]. By detecting changes in light or sound waves as they interact with blood components, these sensors can continuously monitor variations in the platelet count, size, shape, and aggregation properties. This capability of continuous real-time monitoring is particularly advantageous for managing chronic conditions or assessing the long-term effects of therapeutic interventions, offering insights into platelet behaviors that were previously difficult to achieve. However, while these sensors excel in their noninvasive nature and provide continuous data, they may not capture the full depth of information available through more direct, albeit invasive, testing methods. Despite this limitation, optical and acoustic wave sensors represent a significant step forward in the field of platelet-function analysis, offering new possibilities for patient monitoring and treatment optimization.

In Das et al.’s paper [123], the effectiveness of combined ultrasound (US) and photoacoustic (PA) imaging for monitoring blood clot dissolution during microbubble-assisted sonothrombolysis was demonstrated. This technique, which is notable for its ability to analyze blood clot composition through optical absorption sensitivity, was applied to clots in two scenarios: submerged in deionized water and within blood. The study determined that the US and PA signal-to-noise ratio (SNR) values responded differently to sonothrombolysis depending on the environment. Specifically, in deionized water, the US SNR increased marginally, whereas the PA SNR decreased significantly after 30 min. In contrast, in blood, both the US and PA SNR values substantially increased after only 10 min, which was attributed to faster clot degradation facilitated by heparin and better microbubble penetration. These findings suggest that US and PA imaging can effectively track changes in the clot composition, potentially guiding the use of clot-dissolving drugs.

Chen et al. [119] introduced a novel Love-mode surface acoustic wave (SLSAW) sensor for the point-of-care assessment of blood hemostasis, addressing the limitations of traditional coagulation tests such as size, cost, and sample-volume requirements. Operating in the harmonic resonant mode, this disposable plug-and-play SLSAW sensor exhibits a significant frequency shift indicative of blood coagulation and improved signal-to-noise ratios (SNRs). Its effectiveness was validated by comparing its readings with those from standard coagulation tests and TEG analyzers, demonstrating a strong correlation in terms of measuring coagulation times and kinetics. Beyond general coagulation testing, the SLSAW sensor can be adapted for specific platelet function assessments. By adjusting the sensor’s parameters to detect subtle changes in the viscoelastic properties of blood during the early stages of clot formation, researchers can directly evaluate platelet activation and aggregation. Specialized protocols could include the application of platelet-activating factors directly on the sensor’s surface to induce and monitor platelet behavior under controlled conditions. These modifications make the SLSAW sensor a promising tool not only for general hemostasis diagnosis but also for precise, rapid, on-site analysis of platelet function. With its low cost, small size, minimal sample requirement of only 1 μL, and ease of use, the SLSAW sensor represents a significant advancement in the field of hemostasis diagnostics (Figure 4b) [124].

Luo et al. [125] introduced the integrated quasi-static acoustic tweezing thromboelastometry (i-QATT™) method of analyzing blood coagulation in a single drop of blood. Using acoustic radiation to levitate and shape the drop, it measured coagulation-related changes rapidly and accurately within 10 min. i-QATT™ can detect varying coagulable states, factor deficiencies, and the effects of heparin and tPA-induced hyperfibrinolysis. Its ability to provide fast, comprehensive coagulation profiles makes it especially useful for neonates, children, and adults with anemia or blood collection issues and is suitable for both immediate bedside and laboratory applications. Biswas et al. [120] proposed a novel micro-ultrasonic diagnostic method using an all-optical ultrasound-based spectral sensing (AOUSS) technique for the sensitive and quantitative assessment of early stage blood coagulation. The AOUSS technique employed a unique optical transducer for precise frequency-spectral matching and used an empirical mode decomposition (EMD)-based signal processing algorithm to analyze the backscattered signals from a micro-ultrasonic spot generated via laser-focused US. This method enabled the detection of viscoelastic changes in blood during the initial fibrin formation phase of coagulation with high sensitivity and can accurately assess the coagulation stages, with results strongly correlating with those of conventional rheometry (Pearson’s R > 0.95). The ability of AOUSS to detect early clot formation and monitor blood-to-clot transitions offers significant potential for the clinical screening of hypercoagulation and thrombotic disease risk (Figure 4c).

## 5. Supporting Technologies in Platelet Research

### 5.1. High-Throughput Sequencing (HTS)

HTS has significantly impacted the study of inherited platelet disorders (IPDs) and is a powerful tool for uncovering the genetic underpinnings of these conditions. By enabling the rapid and cost-effective sequencing of entire genomes, HTS allows for the precise identification of genetic variations that cause IPDs, laying the groundwork for the development of targeted therapies. This advancement is crucial for tailoring treatments for specific genetic anomalies associated with IPDs, heralding a new era of personalized medicine for the management of platelet-function disorders. The traditional approach of diagnosing IPDs involved a multistep process, as recommended by various guidelines and expert committees. Initially, this included a detailed personal and family history of bleeding; a physical examination for bleeding symptoms and syndromic features; comprehensive laboratory tests for secondary hemostasis defects; the exclusion of von Willebrand disease; and a thorough evaluation of a complete blood cell count (CBC) focusing on platelet count, size, and morphology. Diagnosis typically progresses from simple, nonspecific tests like CBC and blood smear to more specific, complex assessments such as platelet aggregometry and flow cytometry. Despite these methodologies, challenges persist because of the need for large blood samples and the limited availability of advanced tests in specialized laboratories, hindering prompt and accurate IPD diagnosis. However, recent improvements in diagnostic standards and quality control, especially in pediatric patients, have made significant progress in the field [124,126,127,128,129,130,131,132].

Since 2018, HTS has significantly improved our understanding of the genetic basis of platelet-function disorders [119]. This technology has enabled the rapid sequencing of entire genomes, revealing genetic variations associated with a spectrum of platelet-function abnormalities. In addition, Downes et al. [133] utilized a targeted HTS panel to diagnose rare blood disorders in 2396 patients. The panel achieved a diagnostic success rate of 49.2% for various disorders, but only 3.2% for cases of unexplained bleeding. The team identified 745 variants, half of which were novel, including findings in genes recently linked to these conditions. The ability of HTS to uncover these variations with remarkable agility and cost-effectiveness has been pivotal in the move towards personalized medicine, particularly in the development of targeted therapies for specific platelet-related conditions. However, the challenge lies in the complexity of the generated HTS data. The sheer volume of genetic information demands sophisticated bioinformatic tools for interpretation, posing a hurdle in translating these findings into clinical practice. Despite this, the potential of HTS to revolutionize the treatment of platelet-function disorders remains unparalleled, promising a future in which genetic screening can guide more precise and effective interventions.

### 5.2. Mass Spectrometry in Platelet Function Analysis

Mass spectrometry (MS) has been extensively applied in various clinical and research settings to enhance our understanding of platelet functions. Its capability to perform highly sensitive and specific proteomic analysis allows for an in-depth exploration of platelet-related proteins and peptides [134]. This is essential not only for mapping out the complex mechanisms of platelet activation but also for identifying potential biomarkers and monitoring therapeutic interventions. This technology facilitates highly sensitive and specific analyses at the proteomic level, crucial for examining platelet-related proteins and peptides. MS provides detailed molecular insights into platelet activation and the clotting cascade, essential for identifying novel biomarkers and monitoring therapeutic interventions. Recent studies utilizing MS have been pivotal in generating comprehensive human platelet proteomes, revealing nearly 4000 unique proteins [135]. This detailed profiling has improved our understanding of inter-individual variations in platelet functionality, crucial for personalized medicine approaches in treating coagulopathies. MS has also been instrumental in characterizing the protein composition of platelets, thereby enhancing our understanding of platelet granules and the overall hemostatic process in health and disease [136]. Furthermore, the development of targeted proteomics using advanced MS techniques, such as tandem mass spectrometry (MS/MS), has facilitated the quantification of specific proteins related to platelet functions [137]. Techniques like Electrospray Ionization (ESI) and Ion Mobility Spectrometry have refined proteomic analysis, allowing for more detailed and segregated data on platelet proteins [138,139]. These advancements in MS technologies and methodologies have significantly contributed to the field by providing detailed insights at a molecular level, crucial for advancing our understanding of hemostasis and thrombosis. Despite the complexity of MS and the need for specialized equipment, which typically restricts its application to specialized research laboratories, its impact on the field remains unparalleled, promising a future where detailed molecular insights can guide more precise and effective interventions in platelet-function disorders.

### 5.3. Advances in Platelet Research Using AI

The studies in platelet function testing leverage artificial intelligence (AI) to enhance diagnostic accuracy and treatment strategies. In 2012, Flamm et al. [140] explored the use of artificial intelligence to predict platelet phenotypes, specifically focusing on platelet deposition on collagen. This research utilized a microfluidic model to replicate the complex dynamics of platelet behavior. The application of AI in this context underscores its potential in simulating and understanding the intricate biological processes involved in platelet function, which is crucial for advancing diagnostic methodologies in coagulation testing. In 2019, Mishra and Ashraf [141] demonstrated how AI and machine learning (ML) methods like neural networks and natural language processing (NLP) could process large datasets to predict patient responses and identify thromboembolic phenotypes, leading to improved management of thromboembolic disorders (TEDs). In 2020, Yuqi Zhou et al. [142] introduced a convolutional neural network (CNN) that differentiates platelet aggregates by agonist type, a crucial step in understanding platelet behavior in both physiological and pathological contexts. By 2022, Hu, Yongfei et al. [143] described an AI model that predicts the age of stored platelets with over 95% accuracy, using confocal microscopy images to ensure the viability and functionality of platelets for transfusions. Another 2023 study reiterated the capability of AI in optimizing platelet transfusions, enhancing blood storage management, and supporting personalized transfusion strategies [144].

Recent advances in artificial intelligence (AI) offer significant benefits for platelet research, including enhanced diagnostic accuracy, personalized treatments, and increased operational efficiency through the streamlined management and testing of blood products [145]. However, the effectiveness of AI technologies is contingent upon the availability of high-quality, voluminous data, which can be a barrier in less equipped settings. Additionally, the complexity and cost of developing and implementing these systems may restrict their use to well-funded institutions. AI’s decision-making processes, particularly in deep learning, also pose challenges in terms of interpretability, complicating their understanding and acceptance among medical professionals [146]. Looking ahead, integrating AI into routine clinical practice will require further research and refinement, along with improved data collection practices and careful consideration of ethical and regulatory issues to ensure privacy and equity in AI-driven clinical decisions.

## 6. Conclusions and Outlook

The field of platelet-function testing stands on the point of further transformative advancements. The integration of artificial intelligence and machine learning with existing technologies such as HTS promises to overcome the limitations in data interpretation, enabling the extraction of actionable insights from vast genomic datasets with unprecedented precision and efficiency. Similarly, advancements in microfabrication and nanotechnology should reduce the cost and complexity of TEG, ROTEM, and lab-on-a-chip devices, thereby facilitating their wider adoption in various clinical settings. In addition, the continuous evolution of sensor technology, coupled with advancements in wireless communication and data analytics, has been proposed to enhance the capabilities of optical and acoustic wave sensors. These improvements could lead to the development of wearable devices for the real-time ambulatory monitoring of platelet function; this would revolutionize the management of thrombotic disorders and anticoagulant therapy. Furthermore, the convergence of these technologies with the burgeoning field of regenerative medicine, particularly the development of platelet mimetics and bioengineered clotting factors, opens new avenues for therapeutic intervention. Such innovations could provide more effective and safer alternatives to current treatments, reduce the reliance on blood products, and minimize the risks associated with transfusions.

In conclusion, recent advancements in platelet function testing have set a robust foundation for future breakthroughs in hematology. These advancements, driven by artificial intelligence, microfabrication, and improved sensor and communication technologies, have transformed platelet-function testing, enhanced precision and lead to the development of cost-effective, user-friendly diagnostic and real-time monitoring tools. Notably, the potential of wearable devices promises a shift towards personalized and proactive patient care, particularly in managing thrombotic disorders and anticoagulant therapy. Moreover, the combination of these technological advances with regenerative medicine offers new, safer, and more effective treatment alternatives. Despite challenges, the path ahead is filled with opportunities for innovation that will continue to improve patient outcomes and advance the field, emphasizing the crucial role of ongoing innovation and interdisciplinary collaboration in hematology.
